# Assoziation, Erwartungen und Barrieren eines Exoskeletteinsatzes in kleinen mittelständischen Unternehmen

**DOI:** 10.1007/s40664-021-00453-7

**Published:** 2022-01-17

**Authors:** Holger Hoffmann, Imke Pitz, Björn Adomssent, Christoph Russmann

**Affiliations:** Fakultät für Ingenieurwissenschaften und Gesundheit, HAWK Hildesheim/Holzminden/Göttingen, Göttingen, Deutschland

**Keywords:** Industrielles Exoskelett, Arbeitsbedingte muskuloskeletale Erkrankungen, Endnutzer, Herstellende Industrie, Logistik, Industrial exoskeleton, Work-related musculoskeletal disorders, Enduser, Manufacturing industry, Logistics

## Abstract

**Hintergrund:**

Arbeitsbedingte Muskel-Skelett-Erkrankungen (MSE) führen in der herstellenden Industrie zu Krankheitstagen und haben erhebliche wirtschaftliche Folgen für die Unternehmen und die Volkswirtschaft. Exoskelette können den Körper im Umgang mit schwerer Last oder in Zwangshaltungen unterstützen. Besonders in großen Unternehmen der Automobilindustrie werden Exoskelette pilotiert. In kleinen und mittelständischen Unternehmen (KMU) werden Exoskelette bisher jedoch wenig eingesetzt, und ihre Anwendung dort wurde wissenschaftlich bisher kaum untersucht. Ziel dieser Arbeit war es, die Barrieren der Exoskelett-Implementierung und die Erwartungen an deren Einsatz im produzierenden Gewerbe zu ermitteln.

**Methode:**

In sechs produzierenden Unternehmen wurden teilstrukturierte Leitfadeninterviews durchgeführt und analysiert.

**Ergebnisse:**

In den Unternehmen werden vielfältige Tätigkeiten bis an die Belastungsgrenze ausgeführt. Allgemein erwartet man durch die Anwendung von Exoskeletten Arbeitserleichterungen sowie wirtschaftliche Vorteile. Bedenken bestehen hinsichtlich des Einsatzes aufgrund des Kostenfaktors, eines ungewissen Nutzens und mangelnden Tragekomforts. Insbesondere werden mangelnde Kenntnisse über den Effekt eines Exoskeletts deutlich.

**Fazit:**

Die vorgestellten Interviewergebnisse sind ein Schritt im interdisziplinären Prozess der Weiterentwicklung und Implementierung von Exoskeletten in der herstellenden Industrie. Bedenken und Unwissenheit potenzieller Unternehmen und Anwender müssen adressiert werden, auch um eine hohe Nutzerakzeptanz zu schaffen. Folgestudien, die die Ermittlung des Bedarfs mit einer besseren Trennschärfe erheben, könnten weitere Erkenntnisse liefern.

## Abstract (en)

**Background:**

In the manufacturing industry, work-related musculoskeletal disorders (MSD) result in sick days and have substantial economic consequences for the enterprise and the national economy. Exoskeletons can support the body when handling heavy loads and enduring enforced postures. Exoskeletons are being piloted particularly in large companies in the automotive industry; however, exoskeletons have so far attracted little interest in small and medium-sized enterprises (SME) and their use has so far barely been scientifically examined. The aim of this work was to determine barriers to exoskeleton implementation and expectations for their use in the manufacturing sector.

**Method:**

Semi-structured guided interviews in six manufacturing companies were carried out and analyzed.

**Results:**

In the enterprises a variety of activities up to the loading limits are carried out. Exoskeletons are generally expected to facilitate work and provide economic advantages. There are concerns with respect to their use due to cost factors, uncertain benefits and wearing discomfort. Particularly uncertainties about the effects of exoskeletons become evident.

**Conclusion:**

The presented interview results are one step in an interdisciplinary process of further developing and implementing exoskeletons in the manufacturing industry. Concerns and unawareness of potential enterprises and users must be addressed, also to achieve a high user acceptance. Further studies that survey the identification of needs with better discriminatory power could provide additional insights.

Im Interreg-Projekt Exskallerate der EU soll die Einführung von Exoskeletten in KMU der Bereiche Bau, Logistik und industrielle Fertigung aus der Nordseeregion (NSR) beschleunigt werden. Trotz noch geringer Studienlage zur Effektivität von Exoskeletten in der Industrie kommen diese schon jetzt in größeren Industrieunternehmen, wo manuelle Materialhandhabungen ausgeübt werden, in Pilotprojekten zum Einsatz [18]. So werden in Studien, aus größeren Betrieben der Automobilindustrie, der Großindustrie sowie Laboren, Exoskelette bereits pilotiert und getestet [17, 27, 29, 31]. In KMU der herstellenden Industrie werden ebenfalls schwere körperliche Tätigkeiten ausgeführt und das Risiko für MSE ist in diesen – im Vergleich zu größeren Unternehmen – sogar erhöht [8, 29, 30]. Dennoch mangelt es bisher an Implementierungsstudien von Exoskeletten in KMU.

Die Ergonomisierung von Arbeitsplätzen ist in der Regel mit großem finanziellem Aufwand verbunden, den KMU oftmals nicht leisten können. Exoskelette könnte diesen Aufwand der Arbeitsplatzumstellung senken [29]. Um das Ziel der Einführung von Exoskeletten in KMU des NSR zu unterstützen, wurden zunächst Erwartungen, mögliche Barrieren und Assoziationen mit Exoskeletten in eben dieser Zielgruppe abgefragt.

## Theoretischer Hintergrund und Fragestellung

Im produzierenden Gewerbe und der Logistik nehmen arbeitsbedingte MSE zu und verursachen eine hohe Zahl an Krankheitstagen sowie körperliche Einschränkungen mit erheblichen wirtschaftlichen Folgen. MSE sind in Deutschland die Ursache für bis zu 27 % aller Produktionsausfälle durch krankheitsbedingte Abwesenheit [3, 34]. 2015 litten 52 % der Arbeitnehmer in Deutschland in den letzten 12 Monaten unter MSE. Als ökonomische Folgen resultieren geminderte Produktivität und höhere Sozialausgaben. Die damit verursachten Kosten belasteten das Gesundheitssystem und die Volkswirtschaft Deutschlands allein in 2016 mit 17,2 Mrd. € [13]. MSE werden durch physisch belastende Tätigkeiten, in denen durch Muskelkraft schwere Lasten bewegt, gehoben oder getragen werden, begünstigt. Erscheinen diese unvermeidbar, sind Unternehmen gesetzlich verpflichtet, technische, organisatorische und Schulungsmaßnahmen anzubieten und, wo nötig, personenbezogene Schutzmaßnahmen zu treffen sowie Schutzausrüstung einzusetzen [6, 7]. Aktive und passive Exoskelette können den Körper mechanisch unterstützen [22, 28] und fallen durch vielseitigen Einsatzzweck unter unterschiedliche dieser Maßnahmenkategorien und den damit einhergehenden sicherheitstechnischen Regularien [11]. Aktive Exoskelette unterscheiden sich von passiven insofern, dass diese einen elektrischen oder pneumatischen Antrieb vorweisen und dadurch größer und schwerer sind, aber generell mehr Unterstützung bieten können als passive. Passive Exoskelette sind durch ihre rein mechanisch, über Federwirkungen oder Expander, generierte Unterstützung leichter und kostengünstiger [36]. Speziell beim Heben, Tragen und Umlagern von Lasten und bei statischen Körperhaltungen wie Überschulterarbeiten können Exoskelette wirksam unterstützen [32]. Durch die Unterstützung reduziert sich die subjektive Belastung der Nutzer, und die Wahrscheinlichkeit, an einer arbeitsbedingten MSE zu erkranken, könnte minimiert werden [15, 17]. Auch objektive Entlastungen konnten nachgewiesen werden. Verschiedene Studien gehen davon aus, dass die muskuläre Belastung bei Belastungsspitzen innerhalb von Aktivitäten durch das Tragen von passiven, den Rücken unterstützenden, Exoskeletten um 10 bis 40 % und bei statischen Haltungen um bis zu 57 % reduziert werden kann [5, 16, 21, 22, 33]. Außerdem wurde eine Verringerung der wahrgenommenen Anstrengung sowie eine Verlängerung der Ausdauerzeiten in statischen Vorwärtsbeugehaltungen festgestellt [1, 5, 33]. Studien mit passiven Schulterexoskeletten haben gezeigt, dass die Aktivierung der Schultermuskulatur für die agonistische Muskulatur bei Überschulterarbeiten um 16 bis 73 % sinkt und auch die wahrgenommene Anstrengung und der Diskomfort reduziert werden [10, 19, 33]. So können Unternehmen die MSE-bedingten Kosten senken, die Produktivität steigern und eine konstant hohe Qualität der Arbeit ermöglichen [10, 14, 35]. Exoskelette werden in großen Betrieben der herstellenden Industrie bereits angewendet, um muskuloskeletale Belastungen der Arbeitnehmer zu reduzieren [17, 23, 37]. Allerdings unterliegt die Ausbreitung und Akzeptanz von Exoskeletten in den Betrieben vielschichtigen Faktoren [22, 26]. So bilden ein geringer Tragekomfort, die teilweise noch einseitige Anwendbarkeit am Arbeitsplatz und eine niedrige Akzeptanz ein entscheidendes Hindernis für die Ausbreitung von Exoskeletten [5, 17, 22].

Kim et al. [20] untersuchten die Sichtweise der US-amerikanischen Bauindustrie (Baugewerbe) zu dem Einsatz und Nutzen von Exoskeletten als einen ersten Schritt zur Implementierung. Es wurden die Unsicherheiten über den Gebrauchswert, die Kosten, den Nutzen und die Sicherheit der Arbeiter bei Benutzung, aber auch die Hoffnung auf gesteigerte Produktivität, verminderte Gesundheitsgefährdung sowie gesteigerte Motivation deutlich [20, 26]. Exoskelette sollen auch im Rahmen des Projektes Exskallerate in KMU der NSR eingeführt werden, und als erster Schritt einer erfolgreichen Implementierung sollen Informationen über die Erwartungen und mögliche Barrieren dieser Zielgruppe generiert werden. Um Aussagen über Assoziationen zu Exoskeletten im Rahmen der Akzeptanz für den Einsatz dieser bei KMU in der NSR zu erhalten, wurden daher im Zeitraum Dezember 2020 bis Januar 2021 Ansprechpartner aus sechs KMU interviewt. Aus diesen Ergebnissen soll ein erster Überblick über die Barrieren und Erwartungen eines Exoskeletteinsatzes geschaffen werden.

## Methode

Es wurden teilstrukturierte Interviews geführt. Über ein Regionalmanagement-Unternehmen wurden aus der Region Nordhessen und Südniedersachsen Unternehmen aus der Bau- und Logistikbranche kontaktiert und nach Rückmeldung zur Projektteilnahme eingeladen. Mit einem Teil dieser Unternehmen sollten teilstrukturierte Interviews geführt werden. Von allen Teilnehmenden lagen zu Beginn der Interviews Einwilligungs- und Datenschutzerklärung von Ansprechpartner*innen der Unternehmen vor. Es wurden Fragen für ein teilstrukturiertes Leitfadeninterview entwickelt. Die Fragen wurden so konzipiert, dass Antworten zum Wissensstand, zu Assoziationen über Exoskelette und mögliche Barrieren für die Implementierung von Exoskeletten gegeben werden konnten. Nach einer Literatursichtung zu möglichen Antworten auf die genannten Fragen wurden Antwortkategorien gebildet und die Fragen gebildet. Die Ergebnisse von Kim et al. [20] halfen unter anderem (z. B. [2, 12, 29]) bei der Kategorien- und Fragenentwicklung. Die Befragungen fanden durch einen Mitarbeiter des Regionalmanagement-Unternehmens unter Verwendung einer Software für Videokonferenzen statt. Die Transkription der Audiodateien erfolgte durch eine wissenschaftliche Mitarbeiterin. Im Anschluss wurden die Transkripte von zwei Personen unabhängig voneinander analysiert und ausgewertet. Die inhaltliche Analyse fand nach dem von Mayring beschriebenen Verfahren zur Auswertung von teilstrukturierten Interviews statt [24]. Es wurden Generalisierungen und Reduktionen der Antworten durchgeführt. Diese wurden dann den bestehenden Kategorien zugeordnet. Bei Bedarf wurden neue Kategorien und Subkategorien gebildet. In Tab. 2 sind alle Fragen des Leitfadeninterviews aufgelistet sowie alle Kategorien aufgezeigt. Es werden auch Beispiele für die Zuordnung deutlich gemacht. Durch eine Regionalmanagement-Gesellschaft wurde zunächst eine Liste der KMU nach den Projektvorgaben erstellt. Diese Unternehmen wurden zu einem Workshop eingeladen. Im Rahmen dieses Workshops sollten die Interviews durchgeführt werden. Aufgrund der COVID-19-Pandemie konnte dieser nicht stattfinden. Die 18 interessierten Unternehmensvertreter*innen wurden daraufhin per E‑Mail angefragt, ob sie bereit sind, an einem Interview teilzunehmen. Auf Basis der Anfrage haben sich sechs Betriebe zu einem Interview bereiterklärt. Jedes der sechs Unternehmen hat eine*n Ansprechpartner*in zur Verfügung gestellt, der*die die Interviewfragen stellvertretend beantwortet hat. Die Positionen dieser Ansprechpartner*innen im jeweiligen Unternehmen und die Branche des Unternehmens ist Tab. 1 zu entnehmen. Im Folgenden wird von den Antworten der Unternehmen gesprochen; hier ist zu beachten, dass es sich jeweils um die Antworten der stellvertretenden Ansprechpartner handelt.

**Table 1:** 

Branche	Position
*Logistik*	Betriebsleitung Operation
*Lagertechnik*	Geschäftsführung
*Stahlhochbau*	Geschäftsführung
*Baubranche*	Mitarbeiter*in Marketing
*Baunebengewerbe*	Business Development Manager*in
*Produzierendes Gewerbe*	Fachkraft für Arbeitssicherheit

**Table 2:** 

Frage	Kategorie	Subkategorien	Beispiele
*1. Welche schweren körperlichen Tätigkeiten, Bewegungsabläufe werden in Ihrem Betrieb durchgeführt?*	Handhabung schwerer Materialien	–	Allg. Handling großer schwerer Materialien
			Allg. repetitive manuelle schwere körperliche Tätigkeiten
			Handling vibrierender, schwerer Werkzeuge
			Schaufeln, Ausheben, Schweißen
		Heben	Schweres repetitives Heben
			Überkopfheben, Absenken
			Heben ohne Bücken
		Heben mit Rotation	Asymmetrisches Heben
		Tragen	Tragen schwerer Last
		Schieben	Schieben von schweren Lasten
	Belastungsspitzen	–	Hohes Arbeitsaufkommen saisonal
			Treppensteigen
	Zwangshaltungen	–	Repetitiv, kurzweilig Zwangshaltungen
			Zwangshaltungen längerer Dauer
			Gebeugte Haltung
			Kniende Haltung
			Kauernde Haltung
			Liegende Haltung
			Überkopfarbeit
			Steharbeit
	Bereits getroffene arbeitserleichternde Maßnahmen	–	Scherenhubtische
			Stationswechsel
			Aktives Exoskelett
			Geneigte Regalebenen
			Höhenverstellbares Regal
			Wendomat
			Robotik
			Kräne
			Lastenaufzüge
			Rollentische
*2. Welche Körperbereiche erfordern Unterstützung?*	Obere Extremität	–	Arme
	Rücken	–	Wirbelsäule
	Untere Extremität	–	Knie
	Ganzer Körper	–	Muskel-Skelett System
*3. Für welche Tätigkeiten beabsichtigen Sie ggf. ein Exoskelett einzusetzen?*	Umgang schwerer Last	–	Schaufeln
			Tragen
			Repetitives Heben
			Asymmetrisches Heben
	Zwangshaltungen	–	Statisches Halten
	Vibrationsbelastung	–	Montagearbeit
	Wenn technische Hilfsmittel ausgereift sind	–	Arbeitsplatz ist maximal ergonomisch aufgebaut
*4. Hatten Sie schon im beruflichen Kontext Erfahrungen mit Exoskeletten?*	Ja	–	Ein querschnittsgelähmter ehemaliger MA kann damit gehen
			Vorführung durch Hersteller
	Nein	–	Kontaktaufnahme zu Hersteller, hat sich verlaufen
			Projekt mit Exo war geplant, hat sich verlaufen
*5. Was verbinden Sie mit dem Begriff Exoskelett?*	Positiv	Unterstützung	Arbeitserleichterung um die Hälfte
		Gesundheit	Schonung
			Prävention
		Motivation	Mehr Freude bei der Arbeit
		Marketing	Zukunftsperspektive des Jobs
		Neugier	Ob es dadurch tatsächlich eine Entlastung gibt?
	Negativ	Kosten-Nutzen nicht erfüllt	Hohe (Folge‑)Kosten
		Kein/geringer/ungewisser Nutzen	Krankenquote wird dadurch nicht verringert
		Nutzerakzeptanz niedrig	Diskomfort für Mitarbeiter
	Allgemein	Externes Skelett	Aufgesetztes Skelett, Unterstützung von außen
		Roboter/Maschine/Gerät	Passive, aktive Unterstützung (der Extremitäten)
*6. Sammeln Sie Eigenschaften/Merkmale, die Ihnen zum Thema Exoskelette einfallen*	Aktive und passive Exoskelette	–	Es gibt angetriebene und nicht angetriebene
	Einfluss auf Gesundheit	Prävention	Reduzierter Krankenstand durch Hilfsmittel
	Unterstützung	Arbeitserleichterung	Entlastung für den MA
	Vorteil	–	Andere Hilfsmittel oft störend und nicht genutzt. Bei Exo größere Bereitschaft, weil es direkt am Körper getragen wird
		Ökonomisch, produktiv	Menschliche Ressourcen werden geschont, es kann schneller gearbeitet werden
	Innovatives Hilfsmittel	–	Neuartigkeit steigert Nutzerbereitschaft
	Motivation Mitarbeiter	–	Mehr Freude bei der Arbeit
	Nutzerakzeptanz	Hygiene	Schwitzen
		Diskomfort	Hohes Gewicht; es muss leicht sein
		Arbeitssicherheit	Es muss den Arbeitsschutzrichtlinien entsprechen
		Flexibilität	Es muss flexibel sein
		Optik	Es sieht abschreckend aus
		Unwissenheit über Nutzen, Vorteil	MA meinen, sie brauchen es im Moment nicht; Wissen über Exo fehlt
	Langlebigkeit	–	Es sollte nicht sofort kaputt gehen
	Kostenfaktor	–	Der hohe Kostenfaktor ist der große Haken an der Sache
*7. Mit welchem Ziel/mit welcher Absicht möchten Sie ein Exoskelett in Ihrem Betrieb einsetzen?*	Gesundheit	Krankenquote senken	Krankenquote sollte gesenkt werden
		Prävention	Spätere Schäden, im Alter, vermeiden
	Unterstützung	Erleichterung/Entlastung/Hilfe	Wir glauben daran, dass es den MA entlastet
	Vorteil	Ökonomisch positiv	Geringerer Krankenstand ist positiv für Kosten
		Zukunftsorientiert	Attraktivitätssteigerung der Berufe
		Arbeitsakzeptanz	Mehr Freude bei der Arbeit, Motivationssteigerung
	Ergänzung zu unterstützenden Maßnahmen	–	Ergänzung, wo wir mit technischen Mitteln am Ende angekommen sind
*8. Welche Stärken hat Ihrer Meinung nach der Einsatz von Exoskeletten?*	Unterstützung	Entlastung	Des Körpers, der Gelenke; Kraftunterstützung
	Gesundheit	Prävention	Vorsorge für das Alter
	Innovativ/zukunftsorientiert	–	Neuartigkeit
	Ökonomisch positiv	–	Schnelleres Arbeiten, weniger Krankheitstage
	Motivation Mitarbeiter	–	Mehr Freude bei der Arbeit
	Unwissenheit	Nutzen	Keine Erfahrung
*9. Welche Schwächen hat Ihrer Meinung nach der Einsatz von Exoskeletten?*	Kosten	–	Anschaffung lässt sich einfach nicht rechnen
		Folgekosten	Weitere Anschaffungen zum reinen Exo nötig
	Nachteil	Ökonomisch	Hilft nicht im operativen Geschäft, MA müssen weiter rotiert werden zw. Stationen
	Nutzerakzeptanz	Bewegungseinschränkung	Arbeitsbehinderung, da keine spontanen Bewegungen möglich
		Flexibilität	Läuft noch nicht flüssig in Bewegungen, passt sich nicht an
		Gewicht	Hohes Eigengewicht
		Tragekomfort	Diskomfort
		Optik	Nicht ausgereift, sieht noch eigenartig aus
		Hygiene	Schwitzen
	Entwicklungsstand	–	Noch nicht ausgereift
	Unwissenheit	Nutzen	Keine Erfahrung
		Funktion	Keine Erfahrung
*10. Was müssten Sie über den Effekt von Exoskeletten wissen, um deren Einsatz in Ihrem Unternehmen zu befürworten?*	Allgemein	Nutzen	Für MA, für Unternehmen
	Funktion	Leistung	Wie viel Entlastung, ein Beispiel
		Effektivität	Vorteil durch Exo
		Einfluss MA	Schaden durch Exo für MA
		Mechanische Funktion	Erklärung über mechanische Funktionsweise; dynamische/statische Anwendung
	Vorteile ökonomisch	Förderung	–
		Marketing	Bringt es Vorteil für Ansehen des Berufes, des Unternehmens
		Kosten-Nutzen-Faktor	Produktionssteigerung möglich durch Anschaffung; frühe Amortisation
	Nachteile ökonomisch	Kosten	Anschaffung
		Folgekosten	Versicherung, Pflege
		Wartungskosten	Reparaturen, Ersatzteile; Garantie
	Einsatzbereich	Anwendbarkeit auf mehrere Nutzer	Grundgerüst, das geteilt werden kann durch individuelle Erweiterungen
		Bedienbarkeit	Können selbst Änderungen vorgenommen werden
		Grenzen	Wo darf es nicht angewendet werden, draußen/drinnen
		Usability	Vielfältigkeit
	Akzeptanz	–	Wie nehmen MA das Exo an?
	Langzeitwirkung	–	Für MA, für Unternehmen
*11. Kennen Sie die Bestimmungen für den Einsatz von Exoskeletten bzgl. Gesundheitsschutz und Arbeitssicherheit?*	Ja	–	–
	Nein	–	Nein

*Exo* Exoskelett; *MA* Mitarbeiter*in

Im nachfolgenden Abschnitt der Ergebnisse werden diese Antworten dargestellt.

## Ergebnisse

Die teilnehmenden Unternehmen kommen aus den Branchen: Logistik, Lagertechnik, Stahlhochbau und Bau sowie dem Baunebengewerbe und dem allgemein produzierenden Gewerbe (Tab. 1). In allen befragten Unternehmen werden Tätigkeiten ausgeführt, die unter die Lastenhandhabungsverordnung [6] fallen und Tätigkeiten, für die nach dem Arbeitsschutzgesetz [7] geeignete, schützende Maßnahmen zu treffen sind. Erfahrungen mit Exoskeletten im beruflichen Kontext hatten drei der Befragten. Die Erfahrung reicht von Vorführungen eines Herstellers, über den rehabilitativen Kontakt bis hin zum Einsatz eines aktiven Exoskeletts im Unternehmen. Die übrigen Befragten haben keinen Kontakt mit Exoskeletten gehabt, waren jedoch teilweise bereits an Projekten interessiert (Frage 4 in Tab. 2).

Alle befragten Unternehmen berichten von Handhabungen schwerer Materialien, die das Tragen und Heben mit und ohne Rotation beinhalten. Es finden hohe Frequenzen des Arbeitsaufkommens, saisonal sowie auch während des Arbeitsalltags statt, es müssen repetitive und auch ausdauernde Positionen gehalten werden, und es kommt zu Überkopfarbeit. Die Unternehmen haben bereits arbeitserleichternde Maßnahmen getroffen, die technischer und organisatorischer Herkunft sind (Frage 1 in Tab. 2).

Unterstützt werden sollen die obere, untere Extremität oder der Rücken, und zu unterstützende Tätigkeiten sind wiederholendes Heben, statische Haltungen und das Tragen. Vor allem sollen Exoskelette dann eine Unterstützung leisten, wenn technische Hilfsmittel ausgereizt sind (Frage 2 und 3 in Tab. 2).

Der Begriff Exoskelett ruft sowohl positive, negative und neutrale Assoziationen hervor. Alle Nennungen sind unter Frage 5 in Tab. 2 ersichtlich. Die meisten Antworten werden der positiven Kategorie zugeordnet. Neben der Prävention, Unterstützung durch Arbeitserleichterung, einer allgemein gesteigerten Gesundheit durch Schonung wird eine positiv beeinflusste Arbeitsmotivation erwartet. Negative Antworten beschreiben den Kosten-Nutzen-Aspekt, mit hohen Anschaffungskosten und Folgekosten, einer schlechten Nutzerakzeptanz aufgrund eines niedrigen Tragekomforts, und es wird ein geringer sowie ungewisser Nutzen mit dem Begriff Exoskelett verbunden. Allgemein wird ein Exoskelett von allen sechs Betrieben als ein externes Skelett, angesehen, welches sowohl aktiv als auch passiv bei Tätigkeiten unterstützt.

Zu der Frage der vermuteten Eigenschaften und Merkmale von Exoskeletten wurden Merkmale, die die Nutzerakzeptanz des Exoskeletts betreffen, wie Hygiene und Flexibilität und auch Einflüsse auf die Gesundheit, wie ein reduzierter Krankenstand genannt (Frage 6 in Tab. 2). Dem Exoskelett werden Vorteile gegenüber anderen Hilfsmitteln zugesprochen und auch ökonomische Vorteile durch Produktionssteigerungen. Es wird als arbeitsunterstützend für den Mitarbeiter und als motivationsfördernd gesehen. Exoskelette sollten sich durch Langlebigkeit auszeichnen. Des Weiteren wird Exoskeletten ein hoher Kostenfaktor zugesprochen.

Vor allem möchten Unternehmen durch den Einsatz eines Exoskeletts allgemein die Gesundheit fördern, Prävention betreiben und ebenso eine Erleichterung und Unterstützung der Arbeitsbelastung für ihre Mitarbeiter schaffen. Die Betriebe erwarten einen Vorteil, der sich ökonomisch zeigt, die Attraktivität des Berufes steigert und die Arbeitsakzeptanz erhöht. Es soll ergänzend zu bereits vorhandenen unterstützenden Maßnahmen ein Exoskelett zum Einsatz kommen (vgl. Frage 7 in Tab. 2).

Zu den Stärken können zwei Unternehmen – aufgrund mangelnder Erfahrung und Kenntnisse – nicht antworten und verweisen auf die oben wiedergegebenen Absichten. Die vier übrigen Betriebe sehen die durch das Exoskelett geleistete Unterstützung als Stärke, ebenso wie die Prävention und Innovativität und bspw. einen ökonomischen Vorteil (Frage 8 in Tab. 2). Bei den Schwächen werden unterschiedliche Punkte angesprochen (Frage 9 in Tab. 2). Es werden vor allem Schwächen im Bereich der Nutzerakzeptanz erwartet. Es wird eine Bewegungseinschränkung und Arbeitsbehinderung erwartet, ein hohes Eigengewicht und der Tragekomfort wird insgesamt als Schwäche angesehen. Auch wird ein hoher Kostenfaktor mit ökonomischem Nachteil genannt.

Bisher können die sechs befragten Unternehmen einem Einsatz noch nicht befürwortend entgegenstehen, da mangelnde Kenntnisse über das Thema Exoskelett bestehen. Es werden eine Aufklärung über die genaue Funktion eines Exoskeletts im Hinblick auf dessen mögliche Leistung und Effektivität erbeten und Wissen über den Einsatzbereich sowie Akzeptanz gefordert. Vor- und Nachteile über den Einsatz aus ökonomischer Sicht, wie Kosten-Nutzen-Faktoren, Förderungen aber auch Folgekosten und Wartungskosten werden erbeten (Frage 10 in Tab. 2).

## Diskussion

Es wurden anhand der Interviews Erwartungen an Exoskelette, Assoziationen und mögliche Barrieren von KMU der NSR aus den Bereichen der Logistik, dem Baugewerbe und der herstellenden Industrie aus den Leitfadeninterviews abgeleitet. Aus diesen Ergebnissen sollen mögliche Barrieren für eine erfolgreiche Implementierung von Exoskeletten adressiert werden.

In allen beteiligten Branchen werden unterschiedliche Tätigkeiten ausgeführt, ein Anwendungspotenzial von Exoskeletten wurde aus allen Interviews deutlich. So werden in KMU vielfältige Tätigkeiten und sich ändernde Arbeitsschritte durchgeführt (Tab. 2). Exoskelette unterstützen bisher meist eine Tätigkeit, schränken jedoch eine andere Tätigkeit ein [15]. Ist die Tätigkeitspalette vielfältig und wechselnd, können sich somit Probleme in Anwendung und Nutzen von Exoskeletten ergeben. Die Akzeptanz dieser Unterstützung durch die Mitarbeiter ist demnach meist niedrig, da die wahrgenommene Einschränkung der Unterstützungswahrnehmung überwiegt [1, 15]. Im Baugewerbe kommen zudem erschwerte Arbeitsplatzumfelder hinzu. Grenzen der Einsatzbedingungen und Sicherheitsfaktoren des Einsatzes eines Exoskeletts unter solchen Bedingungen sind bisher nicht erforscht [18].

Es ergeben sich hier Hinweise, dass eine generelle Unwissenheit über die Effektivität von Exoskeletten und deren möglichen auch ökonomischen Vorteilen besteht. Eine neue Technologie kann jedoch nur dann akzeptiert werden, wenn gesteigertes Wissen über den positiven Nutzen gegeben ist [12, 18, 25]. Ebenso muss ein Exoskelett zu den Arbeitsbedingungen, dem Arbeitsumfeld und den erwünschten Resultaten passend ausgewählt werden, da die subjektive Wahrnehmung um die Effektivität des Exoskeletts und die Akzeptanz für dessen Verwendung dadurch maßgeblich bestimmt werden [10]. Keinem der Ansprechpartner sind Bestimmungen für den Einsatz von Exoskeletten im Hinblick auf den Gesundheitsschutz und die Arbeitssicherheit geläufig (Frage 11 Tab. 2). Nach diesen Bestimmungen müssen Gefährdungsbeurteilungen des Einsatzes am Arbeitsplatz, erforderliche Schutzmaßnahmen und Einweisungen erfolgen [11].

Eine Herausforderung bei der Datenaufnahme war die geringe Reaktion der KMU auf die Einladungen zur Teilnahme an den Interviews. Die geringe Rücklaufquote führte zu einem kleinen Erhebungskollektiv und könnte ein Zeichen für mangelnde Ressourcen und geringe Kenntnisse über den Nutzen von Exoskeletten in den Unternehmen sein. Es könnte auch ein mangelndes Interesse bei den angefragten Unternehmen vorliegen. Aus den hier gewonnenen Erkenntnissen kann abgeleitet werden, dass die Erwartungen an den Nutzen von Exoskeletten zum einen hoch sind, aber auch Unwissenheit und Skepsis gegenüber dem Nutzen bestehen. Werden diese Punkte nicht adressiert, kann keine erfolgreiche Implementierung erfolgen, da keine Akzeptanz vorherrscht. Dieses Problem einer fehlenden Akzeptanz zeigt sich hier in den häufigen negativen Assoziationen eines Interviewpartners, in dessen KMU bereits ein aktives Exoskelett angeschafft wurde. Das aktive Exoskelett wird von den Mitarbeitern, durch fehlenden Tragekomfort, nicht angewandt, somit ist der Kosten-Nutzen-Aspekt nicht erfüllt.

In den Interviews fällt der oft genannte Kosten-Nutzen-Faktor auf, der für Entscheidungsträger des Unternehmens ein weiterer entscheidender Faktor zur Akzeptanz in den Unternehmen ist [9]. So scheiterte die Anschaffung von Exoskeletten bei zwei Interviewpartnern an den Anschaffungskosten, bei einem weiteren deckt der erwartete Nutzenfaktor des Einsatzes nicht die damit verbundenen Kosten. Die Unternehmen scheinen generell offen gegenüber einer Möglichkeit der weiteren Unterstützung ihrer Mitarbeiter. Dies wird durch die Antworten zu den Absichten des Einsatzes und die generellen Erwartungen, Prävention und Unterstützung deutlich.

Neben den Kosten könnten auch schlechte Erfahrungen mit technischen Maßnahmen wie festen Hebevorrichtungen ein Grund für mangelnde Akzeptanz sein, die sich aufgrund von Mehraufwand und Inflexibilität nicht durchsetzen konnten. Werden bereits angeschaffte technische Hilfsmittel nicht angewandt, wird zusammen mit mangelndem Wissen keine Akzeptanz für ein neues Hilfsmittel gewährleistet sein. Liegen wiederum positive Erfahrungen vor, wie in einem befragten KMU über ein höhenverstellbares Regal, welches die Arbeitsmotivation steigerte und den Krankenstand mindern konnte, zeigt sich deutlich ein positiver Effekt und eine hohe Nutzerakzeptanz gegenüber neuen Technologien. Insgesamt fällt trotz einiger beschriebener Bedenken auf, dass die Mehrzahl der Assoziationen mit dem Begriff Exoskelett, bei fehlenden Kenntnissen über die genaue Funktion, positiv ist. Es mangelt an Wissen über die Funktionsweise, den Nutzen und die Effekte von Exoskeletten, zum anderen scheinen die ökonomischen und sozialen Vorteile unbekannt. Dies führt dazu, dass generell eher Vermutungen über diese gemacht werden. Zu einigen Fragen wurden ähnliche Antworten durch die Interviewpartner gegeben. Dies könnte an einem teilweise fehlenden Wissen der Befragten und an einer zu geringen Trennschärfe der hier entwickelten Fragen liegen. Die hier generierten Ergebnisse können dennoch einen Überblick über den Stand des Wissens und der vorhandenen Erwartungen in den teilnehmenden Unternehmen schaffen und lassen Rückschlüsse auf das größere Kollektiv zu. Die befragten Personen haben alle Positionen in den Unternehmen inne, die nicht in den Nutzerkreis eines Exoskeletts zählen. An den Antworten zum potenziellen Exoskelettgebrauch wird das insofern deutlich, als wiederholend Aspekte eines ökonomischen Vorteils und Kosten-Nutzen-Faktoren genannt werden. Es ist anzunehmen, dass Nutzer eines Exoskeletts einen anderen Blickwinkel mitbringen würden [9]. Für die Interpretation dieser Ergebnisse muss dies beachtet werden, und es sollten in folgenden Interviews potenzielle Endnutzer einbezogen werden. Drei der Ansprechpartner*innen hatten schon Kontakt mit Exoskeletten, jedoch nicht im Rahmen eines Präventionsansatzes der betrieblichen Gesundheitsförderung, weshalb die Ergebnisse mit in die Auswertung aufgenommen wurden. Ein KMU nannte im Interview Erfahrungen mit dem Einsatz eines aktiven Exoskeletts. In dem Interview zeigten sich viele negative Assoziationen. Da die Interviews hier zunächst einen allgemeinen Überblick über den Wissensstand liefern sollten, wurde dieses Unternehmen trotzdem in die Studie eingeschlossen. In zukünftigen Interviews sollten die Interviewpartner weiter nach Erfahrung ohne und mit aktiven und passiven Exoskeletten differenziert werden und in welchen Unternehmensbereichen in welchem Umfang Exoskelette eingesetzt werden.

Barrieren bei der Implementierung von Exoskeletten sind ganz klar die Unwissenheit über Effekte und den Nutzen von Exoskeletten sowie die vielfältigen Tätigkeiten, die in KMU ausgeführt werden müssen. Potenzielle Unternehmen müssen aufgeklärt und ausführliche Tätigkeits- und Arbeitsplatzanalysen müssen generiert werden.

## Limitationen

Es liegen folgende Limitationen vor:

- Es nahmen nur sechs Unternehmen teil. Es sind keine Schlüsse auf die Gesamtheit der KMU in NSR möglich.
- Die Unternehmen stammen aus unterschiedlichen Branchen, mit unterschiedlichen Erfahrungen im Bereich der Exoskelette.
- Verschiedene Fragen haben teilweise gleiche Antworten hervorgebracht, da das Wissen der Befragten nicht ausreichte und auf Vermutungen zurückgegriffen wurde.
- Eine quantitative Analyse ist durch die qualitative Datenerhebung nicht möglich.

## Ausblick

Die erhobenen Daten zeigen, dass mit dem Einsatz von Exoskeletten beim Endnutzer unterschiedliche Assoziationen einhergehen. Die Akzeptanz des Endnutzers scheint eine der wichtigsten Stellschrauben in der Implementierung von Exoskeletten zu sein. Damit sich diese in KMU erhöht, muss Aufklärung betrieben und das Wissen über die nützlichen Effekte von Exoskeletten erweitert werden. Die in der Studie ermittelten Erwartungen der KMU sollten in der Implementierung Beachtung finden. So wünschen sich Unternehmen geringe Kosten, eine einfache Anwendbarkeit, einen wirtschaftlichen und produktiven Vorteil, die Möglichkeit der Prävention und eine Reduktion von Krankheitstagen sowie Motivation bei der Arbeit. Um eine erfolgreiche Einführung von Exoskeletten in KMU zu gewährleisten, muss der Entwicklungsprozess benutzerorientiert ablaufen [4]. Auch, da die Zuhilfenahme eines Exoskeletts häufig als Schwäche des Trägers angesehen und gedeutet wird [1]. Die positiven Effekte des Tragens eines Exoskeletts müssen dem Endnutzer erklärt, gezeigt und durch diesen gespürt werden. Nur mit Daten aus realen Anwendungsbereichen kann eine Anpassung an spezifische Arbeitsumgebungen und erfolgreiche Implementierung angegangen werden. Dieser Implementierungsprozess muss interdisziplinär sein, bestehend aus Nutzern und Interessenvertretern. Das zeigt sich auch in einem Verfahrensmodell, welches technische, kaufmännische und soziale Bereiche umfasst, um Exoskelette in der Industrie erfolgreich einzuführen [14]. Die in der vorliegenden Arbeit vorgestellten Interviewergebnisse sind ein Schritt im interdisziplinären Prozess der Implementierung und Weiterentwicklung von Exoskeletten unter Einbeziehung der Nutzer. Anhand dieser Ergebnisse können Barrieren, wie das fehlende Wissen über den Nutzen des Exoskelett-Einsatzes für Unternehmen der NSR aus der Logistik, dem Bauwesen und der herstellenden Industrie identifiziert und eliminiert werden.

## Fazit für die Praxis

- Erwartungen und Assoziationen mit dem Einsatz von Exoskeletten sind vielfältig.
- Mangelndes Wissen in Unternehmen bezüglich des Kosten-Nutzen-Faktors, der Anwendbarkeit, der Funktion und der Effektivität erschwert die Teilnahme von Unternehmen an Forschungsprojekten zur Implementierung.
- Die Nutzerakzeptanz wird vielschichtig beeinflusst.
- Exoskelette sollten in einem interdisziplinären Prozess, unter Einbindung der Endnutzer, implementiert und deren Nutzen in der Anwendung weiter erforscht werden.
